# Multimodality treatment and survival in primary cardiac malignancies: evidence from competing-risk and machine learning analyses

**DOI:** 10.1007/s12094-026-04322-8

**Published:** 2026-04-01

**Authors:** Md Roungu Ahmmad, Morshed Alam, Michael Baine, Md Tareq Ferdous Khan

**Affiliations:** 1 College of Nursing, USF-Health, University of South Florida, Tampa, FL USA; 2 Merck & Co., Inc., Rahway, NJ USA; 3 Department of Radiation Oncology, University of Nebraska Medical Center, Omaha, NE USA; 4 Department of Public Health Sciences, College of Behavioral, Social and Health Sciences, Clemson University, Clemson, SC USA; 5 Clemson University School of Health Research, Clemson, SC USA

**Keywords:** Primary cardiac malignancies, Competing risks, Treatment modality, Survival analysis, Machine learning

## Abstract

**Background:**

Primary cardiac malignancies are rare and highly aggressive, with limited clinical evidence to guide optimal treatment. This study evaluated the association between multimodal treatments and survival outcomes under competing-risk conditions.

**Methods:**

A retrospective cohort analysis was conducted using SEER-18 data (2000–2018) to identify patients with cardiac malignancies. Overall survival and cause-specific mortality were assessed using Kaplan–Meier and Fine–Gray competing-risk models. Multimodal treatment effects were examined using adjusted regression analyses. Prognostic performance was further evaluated using machine learning methods, including random survival forests and survival trees, with discrimination, calibration, and Brier scores assessed in a validation cohort.

**Results:**

Among 416 patients, angiosarcoma predominated, most had regional/distant disease, surgery and chemotherapy were common, and over 80% experienced either primary cardiac malignancy-specific mortality or other-cause mortality. Chemotherapy plus surgery was associated with lower primary cardiac malignancy-specific mortality (AHR: 0.72; 95% CI 0.54–0.95) and other-cause mortality (AHR: 0.58; 95% CI 0.36–0.93). Surgery with radiotherapy was not significantly associated with primary cardiac malignancy-specific survival but with lower other-cause mortality (AHR: 0.44; 95% CI 0.22–0.87). Compared with surgery alone, multimodal combinations were not significantly associated with primary cardiac malignancy-specific mortality; however, surgery with chemotherapy (AHR: 0.54), surgery with radiation (AHR: 0.29), and triple therapy (AHR: 0.34) were linked to lower other-cause mortality. Regression tree revealed age- and stage-specific heterogeneity, with greater survival likelihood among patients ≤ 65 years with regional/distant disease who underwent surgery.

**Conclusion:**

Surgery, particularly when combined with chemotherapy, was associated with improved outcomes. Benefits varied by age and stage, underscoring the need for individualized multimodal treatment strategies.

## Introduction

Primary cardiac cancer, also known as heart cancer, refers to malignant tumors that develop within the heart tissue and are exceedingly rare, approximately 0.001–0.3% of all cancers [1–4]. Among primary cardiac malignancies, angiosarcoma is the most common subtype in adults, whereas rhabdomyosarcoma occurs more frequently in children [5, 6]. These tumors are highly aggressive and generally associated with a poor prognosis [3, 7]. Even with treatment, survival outcomes remain limited, and disease progression is typically rapid [3, 4]. Delayed diagnosis is common due to the nonspecific nature of early symptoms and the anatomical complexity of the heart, which makes detection challenging [8].

Population-based studies indicate that surgery remains the primary intervention for cancer outcomes, offering a survival advantage, while chemotherapy may also provide clinical benefits [9]. Radiotherapy generally plays a more limited role in treatment for rare cancers [10]. Surgical resection, even when not complete, is associated with better outcomes in some patient groups, and multimodal treatment approaches are often considered when clinically feasible [11, 12]. However, the optimal combination and sequencing of these therapies remain undefined due to the rarity of the disease and limited prospective data [13, 14]. Evidence suggests that integrating systemic therapy with surgery may enhance disease control in certain subgroups, but outcomes vary by tumor histology, stage, and patient characteristics [15–17]. Comprehensive, population-based analyses are essential to delineate treatment effectiveness and guide evidence-based strategies for managing this rare and aggressive cancer.

The influence of age on primary cardiac malignancies is well-documented, whereas sex-based differences in prognosis remain unclear [18, 19]. Moreover, limited evidence exists regarding racial or ethnic disparities in outcomes for patients with primary cardiac tumors [20, 21]. Broader oncology research suggests that minority populations frequently experience delayed diagnosis and reduced access to care, which may also affect patients with cardiac sarcomas [20–22]. Given the rarity and highly aggressive nature of cardiac sarcoma and the absence of standardized treatment guidelines, therapeutic decisions are often based on small case series or institutional experience. Most published studies are single-center, including 1 to 27 cases of malignant primary cardiac tumors spanning over 9 to 49 years, highlighting the rarity and variability of the available data [15, 23–27].

Despite advances in oncologic care, the therapeutic impact of multimodal treatment strategies in patients with primary cardiac malignancies remains poorly understood. Existing studies are constrained by small sample sizes and the lack of integrated analyses that account for demographic, multimodal treatment, and advanced computational factors. To address this gap, this study leverages a large, population-based cancer registry and advanced statistical/machine learning methods to evaluate the association between multimodal treatment and survival outcomes, uncover nonlinear interactions, enhance prognostic precision, and inform evidence-based management for this rare and aggressive cancer.

## Methods

### Study design and sample

**Fig. 1:**
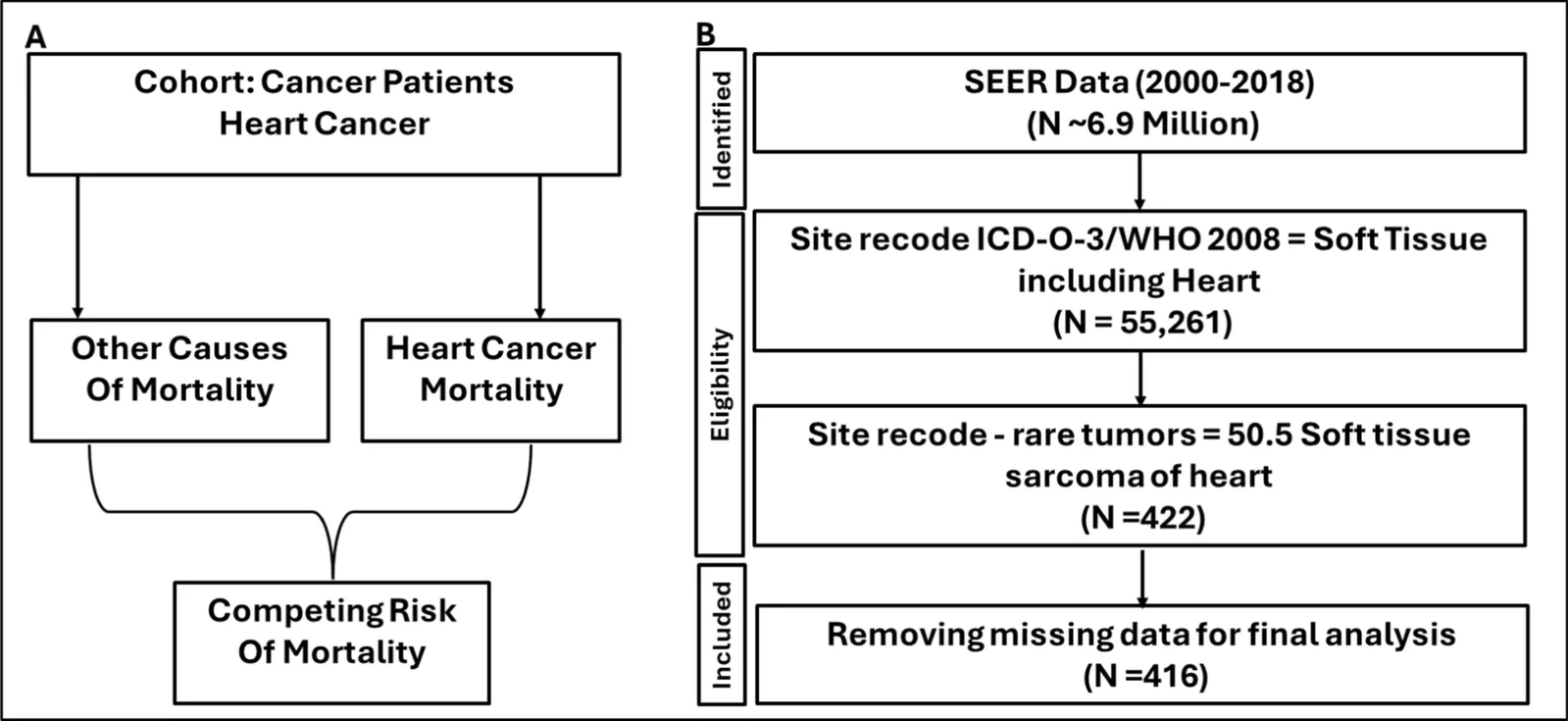
Study design and sample selection process. Panel **A** illustrates the competing-risk framework and Panel **B** depicts the process to derive the analytical sample

This study used a retrospective cohort from the U.S. National Cancer Institute’s Surveillance, Epidemiology, and End Results (SEER-18) registries spanning 2000–2018. The SEER database provides comprehensive, population-based data on cancer incidence and survival, covering about 34.6% of the U.S. population [28]. From an initial dataset of roughly 6.9 million records, eligibility criteria identified cases with complete demographic, clinical, and treatment information. Patients with primary cardiac tumors were identified using the SEER Cause of Death (COD) to Site Recode variable (ICD-O-3/WHO 2008) under the category *“Soft Tissue Including Heart.”* Of 55,261 eligible cases, further restriction to the SEER Rare Tumors List (subsection 50.5), *“Soft Tissue Sarcoma of the Heart,”* yielded the target cohort of 422 patients. After excluding cases with missing or incomplete key variables, the final analytic sample included 416 patients with histologically confirmed primary cardiac malignancies (Fig. 1B). Institutional Review Board (IRB) approval was not required, as this study used de-identified SEER data.

### Primary outcomes

The primary outcomes of interest were overall survival (OS) and cause-specific mortality (CSM). Overall survival was defined as death from any cause during the follow-up period, whereas cause-specific mortality represented death directly attributable to cardiac sarcoma. Deaths from other causes were treated as competing events to account for their potential influence on outcome estimation. Although SEER derives cause-of-death information from death certificates using standardized coding procedures, misclassification may occur, particularly when distinguishing cancer-specific mortality in complex clinical scenarios. This competing-risk framework provided a comprehensive evaluation of patient survival and treatment effects while appropriately distinguishing disease-specific mortality from unrelated deaths in this rare and aggressive malignancy (Fig. 1A). Details of data and variables can be found from SEER website (https://seer.cancer.gov/).

### Key predictors

The primary predictors in this study were surgery, radiotherapy, chemotherapy, and their various combinations. Each treatment modality was coded as a binary variable: “yes” if the patient received the respective treatment in any form and “no” if the patient did not receive any.

Patients were categorized as having received surgery if they underwent any form of tumor resection regardless of the extent of operation. Chemotherapy was classified as “yes” if systemic anticancer treatment, including neoadjuvant, adjuvant, or palliative, was administered. In the SEER data, chemotherapy is originally coded as “yes” versus “no/Unknown,” without details on agents, dose, cycles, or timing; accordingly, we classified chemotherapy as “yes” if recorded as administered and “no” otherwise. For radiotherapy, although multiple categories exist (e.g., beam radiation, radiation not otherwise specified, none/unknown, refused, recommended but unknown if administered), they do not consistently capture dose, fractionation, modality, or treatment intent, and some categories have sparse counts. To ensure analytic stability, and address sparse data, radiotherapy was also dichotomized as “yes” (any form of radiation recorded) versus “no” (none/unknown or refused). These definitions are consistent with prior SEER-based oncology studies of rare cancers [3, 29].

Combinations of these modalities, such as surgery plus chemotherapy, surgery plus radiotherapy, or all three defined as triple therapy, were included to assess potential synergistic or additive effects on survival. This binary and combinatorial classification facilitated comprehensive modeling of treatment-related predictors in predictive analyses.

### Covariates

Explanatory variables were selected based on clinical relevance and prior evidence from cardiac cancer research. Age at diagnosis was treated as a continuous variable to capture its linear relationship with survival outcomes. Sex was categorized as male or female. Race was grouped into three categories (White, Black, and Other), with the “Other” category combining all remaining racial groups due to their small counts. Key clinical variables included cancer stage at presentation (localized, regional, and distant), reflecting disease progression and extent of metastasis. Tumor histology, particularly angiosarcoma (yes/no), was included as a distinct predictor given its aggressive biological behavior and poor prognosis compared with other cardiac tumor subtypes. These variables were included in both statistical and machine learning models to evaluate their independent and joint effects on survival outcomes.

### Analytic approaches

#### Statistical analysis

Descriptive statistics summarized demographic, clinical, and treatment characteristics. Categorical variables were reported as frequencies and percentages, and continuous variables as medians with interquartile ranges (IQRs). Overall survival was estimated using the Kaplan–Meier method and compared across groups with log-rank tests. Within the competing-risk framework, primary cardiac malignancy-specific mortality was the primary outcome, and deaths from other causes were treated as competing events (Fig. 1A). Cumulative incidence functions (CIFs) estimated cause-specific and other-cause mortality, and Fine–Gray sub-distribution hazard models evaluated associations between treatment modalities and mortality outcomes, adjusting for demographic and clinical covariates. Results were expressed as adjusted hazard ratios (AHRs) with 95% confidence intervals (CIs), with *p* < 0.05 indicating statistical significance. Given the observational nature of SEER data, analyses were designed to estimate associations rather than causal effects, recognizing the potential influence of selection bias and unmeasured confounding.

#### Machine learning analysis

To enhance prediction accuracy and capture complex interactions, competing-risk random forest (RF) and random survival forest (RSF) models were applied [30–32]. These tree-based ensemble methods model nonlinear effects and estimate variable importance [31]. Survival regression trees were further developed to classify patients into distinct risk subgroups based on clinical and treatment characteristics, providing interpretable frameworks for individualized survival prediction [33].

#### Validation and performance evaluation

Data was randomly divided into training (70%) and testing (30%) sets. Models were developed using the training data and evaluated on the testing data. Performance was assessed through ROC curves and Area Under the Curve (AUC) for discrimination, calibration plots for agreement between predicted and observed outcomes, and Brier scores for overall prediction error. The ML may be subject to overfitting due to the limited sample size and the number of candidate predictors, and that the reported performance metrics should therefore be interpreted cautiously. All analyses were performed using R software (version 4.5.2; R Foundation for Statistical Computing, Vienna, Austria).

## Results

### Sample characteristics

A total of 416 primary cardiac malignancy patients were included in this study. The median age at diagnosis was 48 years (IQR: 35–62), increasing to 55 years (IQR: 38–67) for those who died from other causes. The cohort comprised 51% females (*n* = 212) and 49% males (*n* = 204). Racially, 78% were White, 12% Black, and 10% other racial groups. At diagnosis, 29% were localized, 31% regional, and 40% distant-stage cancer. Angiosarcoma accounted for 45% of cases. Treatment modalities included radiotherapy (22%), chemotherapy (51%), and surgery (66%), with surgical status missing for two patients. Outcomes showed 68 censored, 247 died from primary cardiac malignancy, and 101 died from other causes (Table 1).

**Table 1:** Demographic and clinical characteristics stratified by survival outcomes of interest

Predictors	Overall (*n* = 416)	Censored (*n* = 68)	Mortality		*p* value
			Primary cardiac malignancies (*n* = 247)	Other causes (*n* = 101)	
Age in years^†^	48 (35, 62)	44 (32, 56)	48 (35, 60)	55 (38, 67)	0.016
Sex					0.15
Female	212 (51%)	37 (54%)	132 (53%)	43 (43%)	
Male	204 (49%)	31 (46%)	115 (47%)	58 (57%)	
Race/ethnicity					0.77
Others	43 (10%)	7 (10%)	25 (10%)	11 (11%)	
Black	50 (12%)	6 (9%)	34 (14%)	10 (10%)	
White	323 (78%)	55 (81%)	188 (76%)	80 (79%)	
Cancer stage					0.039
Localized	106 (29%)	19 (41%)	66 (29%)	21 (22%)	
Regional	114 (31%)	17 (37%)	64 (28%)	33 (35%)	
Distant	149 (40%)	10 (22%)	98 (43%)	41 (43%)	
(Missing)	47	22	19	6	
Angiosarcoma [yes]	188 (45%)	28 (41%)	120 (49%)	40 (40%)	0.24
Radiotherapy [yes]	92 (22%)	16 (24%)	61 (25%)	15 (15%)	0.13
Chemotherapy [yes]	212 (51%)	41 (60%)	131 (53%)	40 (40%)	0.018
Surgery [yes]	274 (66%)	58 (85%)	152 (62%)	64 (64%)	0.001
(Missing)	2	0	1	1	

^†^Continuous variables are presented as median and interquartile range (IQR); categorical variables are presented as frequency and percentage

^†^*p* values were obtained using the Kruskal–Wallis rank-sum test for continuous variables and the chi-squared test for categorical variables

### Survival analysis

**Fig. 2:**
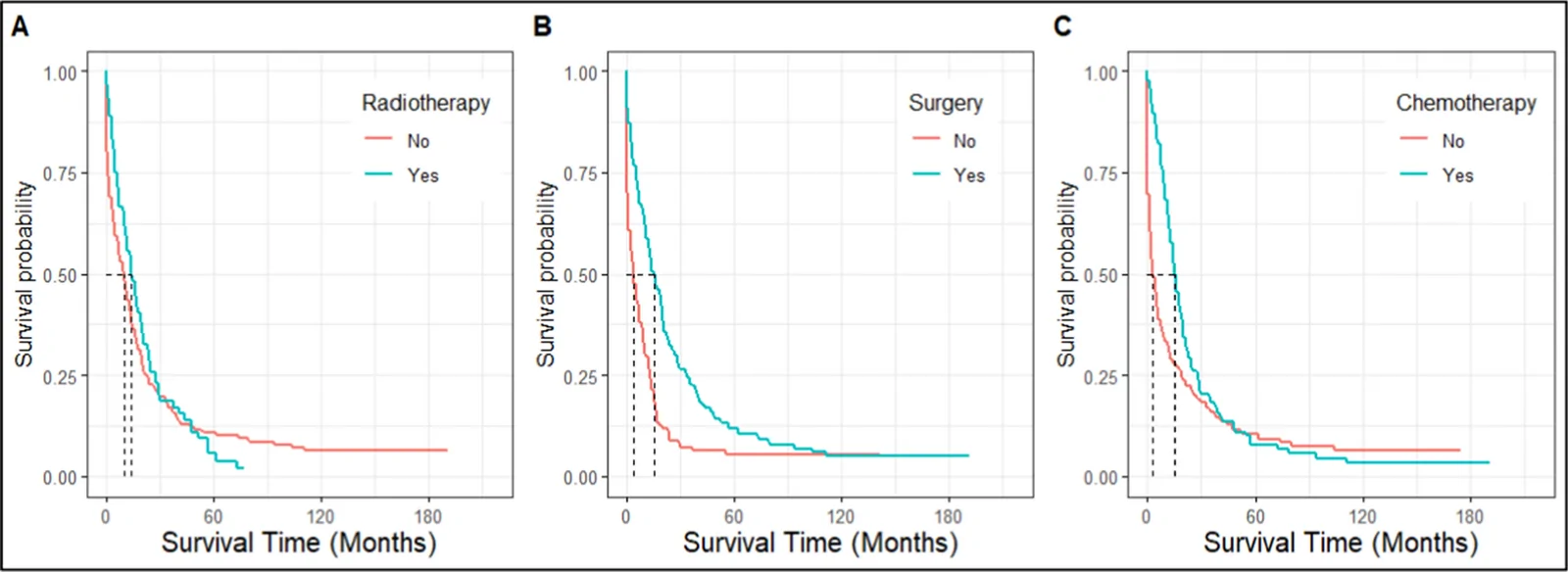
Kaplan–Meier survival curves for all-cause mortality stratified by treatment modalities: Panel **A** for radiotherapy, Panel **B** for surgery, and Panel **C** for chemotherapy

Figure 2 presents univariate Kaplan–Meier overall survival analyses stratified by treatment modalities: (A) radiotherapy, (B) surgery, and (C) chemotherapy. Across all modalities, survival probability declines sharply during the early follow-up period, reflecting high short-term mortality among patients with cardiac cancer. Patients who underwent surgery showed the highest and consistent survival benefit over the follow-up period (Fig. 2B). Radiotherapy and chemotherapy were also associated with higher short-term survival compared to no treatment, although these differences diminished over time (Fig. 2A and C). These findings indicate differences in survival across treatment groups; however, further multivariable analyses are needed to account for potential confounding factors.

**Fig. 3:**
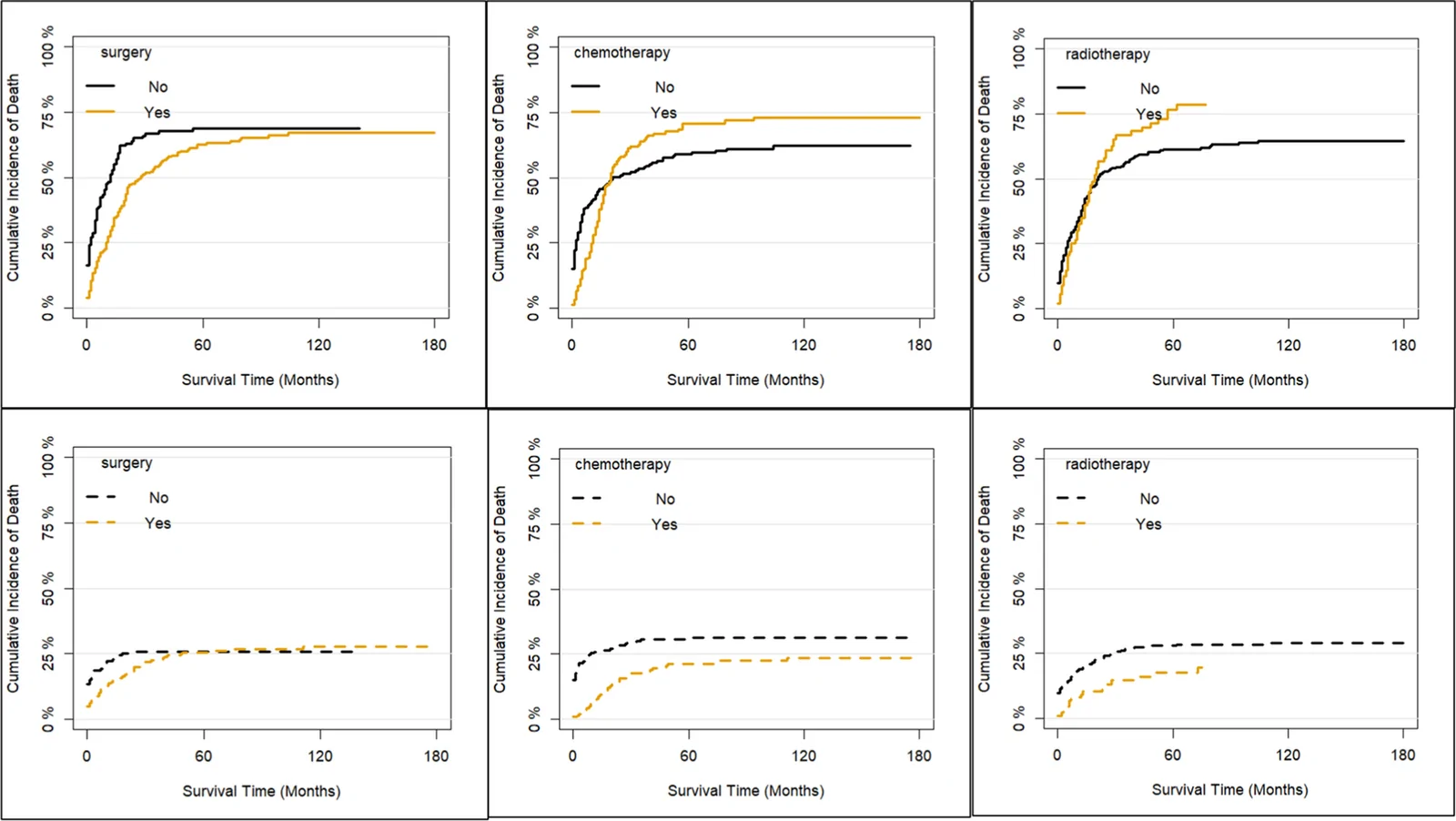
Competing-risk analysis of mortality by treatment modality using cumulative incidence functions. Solid lines represent primary cardiac malignancy-specific mortality, while dashed lines represent mortality from other causes

Competing-risk analyses demonstrate differences in the cumulative incidence of primary cardiac malignancy-specific mortality versus other-cause mortality across treatment groups (Fig. 3). The solid lines represent primary cardiac malignancy-specific mortality, while the dashed lines represent mortality from other causes. Patients who underwent surgical treatment showed lower cumulative incidence of primary cardiac malignancy-specific mortality compared with those who did not receive surgery. However, mortality from other causes did not differ significantly between the two groups over the follow-up period. Chemotherapy was associated with a reduction in primary cardiac malignancy-specific mortality during the first three years of follow-up. After this period, the association with cancer-specific mortality was diminished; however, chemotherapy remained significantly associated with lower mortality from other causes throughout the study period. Radiotherapy showed no substantial difference in primary cardiac malignancy-specific mortality during the initial years of follow-up. Over time, the cumulative incidence of primary cardiac malignancy-related death declined among patients who did not receive radiotherapy, while radiotherapy was associated with lower mortality from other causes across the full follow-up duration.

Table 2 summarizes results of multivariable competing-risk models for primary cardiac malignancy-specific mortality and other-cause mortality. Age was positively associated with cancer-specific mortality (AHR: 1.01, 95% CI: 1.01–1.02) and other-cause mortality (AHR: 1.026, 95% CI: 1.01–1.04). Sex was not statistically significant. Disease stage at diagnosis showed the strongest association: compared to localized stage, regional was associated with a more than twofold higher hazard of other-cause mortality (AHR: 2.36; 95% CI: 1.34–4.15), while distant-stage was associated with a 69% higher hazard of primary cardiac malignancy-specific mortality (AHR: 1.69; 95% CI: 1.18–2.43) and approximately threefold higher for other-cause mortality (AHR: 3.01; 95% CI: 1.68–5.41). Angiosarcoma histology was associated with a 35% higher hazard of primary cardiac malignancy-specific mortality (AHR: 1.35; 95% CI: 1.01–1.82).

**Table 2:** Multivariable competing-risk analysis of primary cardiac malignancy-specific mortality and other-cause mortality

Predictors	AHR (95% CI)	
	Primary cardiac malignancy-specific mortality	Other causes of mortality
Age in years	1.01 [1.01–1.02]	1.03 [1.01–1.04]
Sex (ref. = Female)		
Male	0.84 [0.64–1.10]	1.46 [0.96–2.21]
Race/ethnicity (ref. = Other)		
Black	1.10 [0.64–1.90]	0.80 [0.33–1.95]
White	0.97 [0.62–1.51]	0.79 [0.41–1.51]
Cancer stages (ref. = Localized)		
Regional	1.24 [0.87–1.77]	2.36 [1.34–4.15]
Distant	1.69 [1.18–2.43]	3.01 [1.68–5.41]
Angiosarcoma	1.35 [1.01–1.82]	0.71 [0.44–1.12]
Radiotherapy	0.95 [0.70–1.30]	0.57 [0.32–1.01]
Surgery	0.57 [0.42–0.79]	0.64 [0.39–1.06]
Chemotherapy	0.63 [0.47–0.85]	0.51 [0.32–0.81]

*AHR* adjusted hazard ratios, *CI* confidence interval

Among treatment modalities, surgery and chemotherapy were associated with lower primary cardiac malignancy-specific mortality. Surgery corresponded to a 42% lower hazard of primary cardiac malignancy-specific mortality (AHR: 0.58; 95% CI: 0.42–0.79). Chemotherapy was similarly related to lower hazards of both primary cardiac malignancy-specific mortality (37% reduction, AHR: 0.63, 95% CI: 0.47–0.85) and other-cause mortality (49% reduction, AHR: 0.51, 95% CI: 0.32–0.81). Radiotherapy was not statistically significantly related to primary cardiac malignancy-specific mortality but demonstrated a borderline association with lower other-cause mortality (AHR: 0.57; 95% CI: 0.32–1.01) (Table 2). It is noted here and throughout that the association of treatment modalities with other-cause mortality may reflect selection bias rather than treatment efficacy.

Table 3 presents multivariable competing-risk analyses examining the associations of multimodal treatments with primary cardiac malignancy-specific and other-cause mortality. The reference group included patients who received no therapeutic intervention. The combination of chemotherapy and surgery showed significantly higher survival from both primary cardiac malignancy-specific mortality (AHR: 0.72; 95% CI: 0.54–0.95) and other-cause mortality (AHR: 0.58; 95% CI: 0.36–0.93). Similarly, the combination of radiotherapy and surgery was associated with a 56% lower hazard of other causes of mortality (AHR = 0.44; 95% CI: 0.22–0.87), while no significant association was observed for primary cardiac malignancy-specific mortality. Both radiotherapy plus surgery and triple therapy (radiotherapy, surgery, and chemotherapy) showed lower but insignificant hazards for both mortality outcomes.

**Table 3:** Multivariable analysis of competing-risk of mortality for multimodal treatment combinations among primary cardiac malignancy patients

Therapeutic combination	AHR (95% CI)	
	Primary cardiac malignancy-specific mortality	Other causes of mortality
Radiation + surgery	1.06 [0.75–1.48]	**0.44 [0.22**–**0.87]**
Chemotherapy + surgery	**0.72 [0.54**–** 0.95]**	**0.58 [0.36**–**0.93]**
Chemotherapy + radiation	0.96 [0.68–1.35]	0.59 [0.31–1.12]
Radiation + surgery + chemotherapy	0.99 [0.66–1.47]	0.48 [0.21–1.10]

*AHR* adjusted hazard ratio, *CI* confidence interval; Models were adjusted by age, sex, race, and cancer stages; no therapeutic intervention was considered as the reference for both models

Table 4 summarizes results of multivariable competing-risk models comparing multimodal treatment approaches to the reference group of ‘surgery only’. Across treatment groups, none of the combination therapies showed a statistically significant difference in primary cardiac malignancy-specific mortality relative to surgery alone. However, mortality from other causes was lower among patients receiving additional treatments: surgery plus chemotherapy (AHR: 0.54, 95% CI: 0.29–0.98), surgery plus radiation (AHR: 0.29, 95% CI: 0.08–0.96), and surgery combined with chemotherapy and radiation (AHR: 0.34, 95% CI: 0.14–0.83). All models were adjusted for age, sex, race, cancer stage, and angiosarcoma subtype.

**Table 4:** Multivariable analysis of competing risks of mortality for multimodal treatment combinations among primary cardiac malignancy patients

Therapeutic combination	AHR (95% CI)	
	Primary cardiac malignancy-specific mortality	Other causes of mortality
Surgery + chemotherapy (ref. Surgery only)	0.78 [0.51–1.19]	**0.54 [0.29–0.98]**
Surgery + radiation (ref. Surgery only)	1.32 [0.73–2.38]	**0.29 [0.08–0.96]**
Surgery + chemotherapy + radiation (ref. Surgery only)	1.01 [0.62–1.65]	**0.34 [0.14–0.83]**

‘Surgery only’ was considered as the reference for each model

*AHR* adjusted hazard ratio, *CI* confidence interval; Models were adjusted by age, sex, race, cancer stages, and angiosarcoma

### Machine learning modeling

**Fig. 4:**
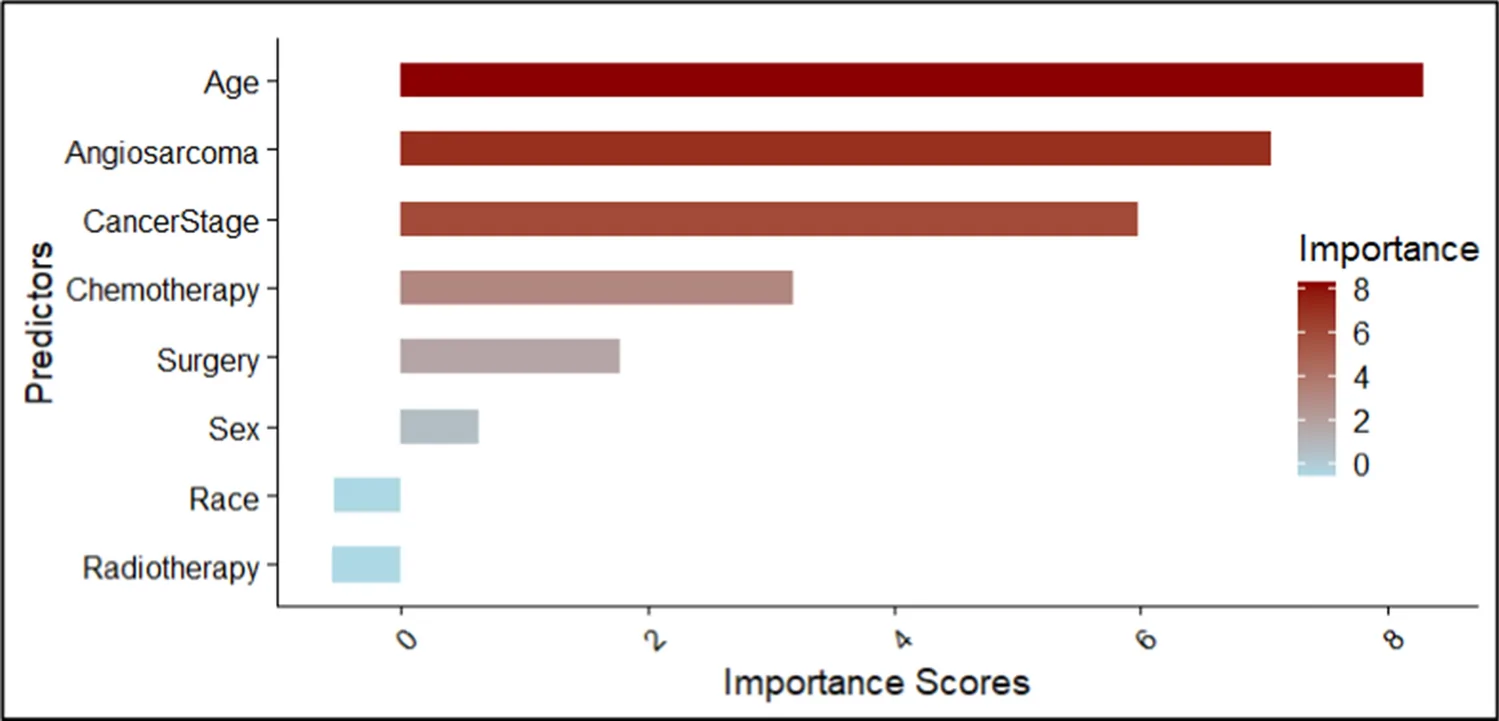
Variable importance scores from the random forest (RF) model for competing-risk of mortality

Figure 4 shows the ranking of variables based on importance scores derived from the random forest (RF) model. Age had the highest importance score (importance score ≈ 8), indicating it contributed most to the prediction model mortality. Angiosarcoma and cancer stage followed closely (importance score ≈ 6,7), reflecting the substantial contributions to mortality prediction within the model. Chemotherapy and surgery showed moderate importance (importance score ≈ 2–6), highlighting their therapeutic relevance. In contrast, sex, race, and radiotherapy had low importance scores, indicating minimal contribution to mortality prediction in this analysis.

**Fig. 5:**
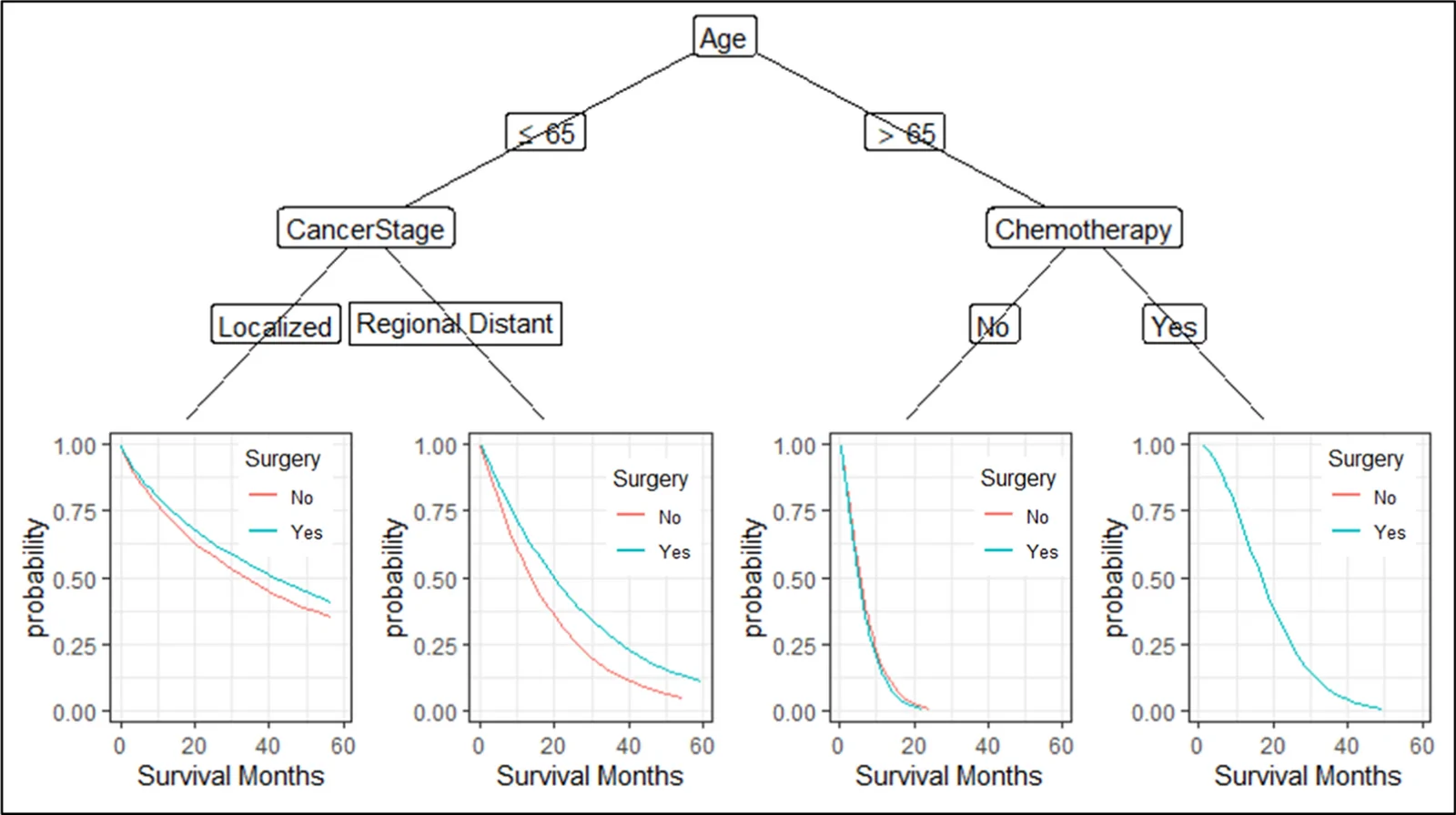
Regression tree from a machine learning competing-risk survival model

Figure 5 presents the regression tree from the machine learning competing-risk model, illustrating how demographic, clinical, and treatment factors were used to stratify survival outcomes. Age was the primary split variable, indicating that it contributed most to survival stratification. Among patients aged ≤ 65 years, cancer stage was the key secondary determinant, with localized disease corresponding to most favorable survival outcomes. Surgical intervention was consistently associated with higher survival probabilities across stages, particularly among patients with localized and regional disease. Among patients aged > 65 years, chemotherapy emerged as the primary stratifying variable. In this subgroup, surgery showed limited differences in survival among patients who did not receive chemotherapy. Among older patients who received chemotherapy, surgery corresponded to slightly higher survival probabilities, although overall survival declined rapidly over time across subgroups.

**Fig. 6:**
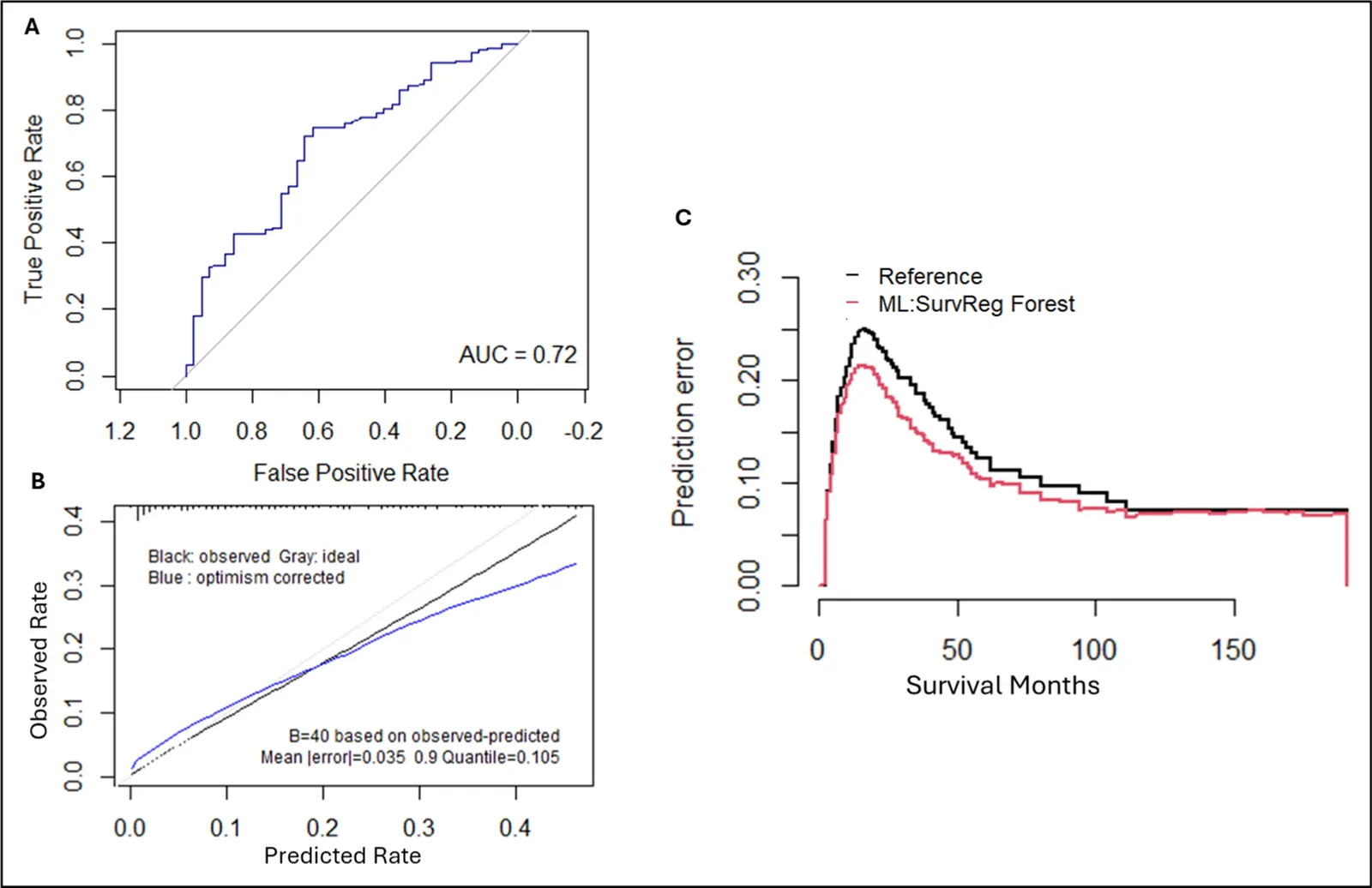
Model performance evaluation on the test dataset. **A** Receiver Operating Characteristic (ROC) curve with area under the curve (AUC) = 0.72 for the machine learning model. **B** Calibration plot comparing predicted versus observed outcomes. **C** Prediction error assessment. The machine learning model was compared with a Cox proportional hazards model, which served as the reference model

The machine learning regression tree model demonstrated solid predictive performance in the test dataset (Fig. 6). The ROC curve (Panel A) yielded an AUC of 0.72, indicating acceptable discriminative ability to distinguish patients with higher versus lower observed mortality risk. The calibration plot (Panel B) showed close alignment between observed and predicted probabilities, with a mean absolute error of 0.035 and a 90th percentile error of 0.105, confirming reasonable calibration. Prediction error analysis (Panel C) showed lower prediction error for the machine learning forest model (gray line) compared to the reference model (black line). Overall, the model demonstrated a balance between discrimination and calibration in the test data, with lower prediction error, indicating stable predictive performance within the sample.

## Discussion

In this population-based study, primary cardiac malignancies, survival patterns were largely associated with age, tumor biology, and disease stage. Surgery and chemotherapy were each associated with significantly higher overall and cause-specific survival, whereas radiotherapy demonstrated limited associations, potentially reflecting its use in selected or palliative cases. Multimodal treatment approaches were associated with lower mortality from other causes compared with surgery alone, but no significant association was observed in primary cardiac malignancy-specific mortality. Machine learning methods, including random survival forests and competing-risk models, provided additional insights into the relative importance of prognostic factors and revealed nonlinear treatment-outcome relationships, and improved individualized mortality prediction for this rare, aggressive cancer.

The early steep decline in survival, even among patients receiving active treatment, reflects the highly aggressive nature of cardiac sarcomas and the challenges in early detection. These tumors often present at advanced stages due to nonspecific symptoms and delayed diagnosis, resulting in rapid disease progression and poor outcomes [17]. Consistent with previous studies, surgical resection was linked to higher survival probabilities, particularly among patients with localized or regionally advanced disease [27, 34, 35]. When technically feasible, complete surgical resection remains the cornerstone of therapy, offering the best opportunity for long-term survival and potential cure [11, 34]. Achieving negative surgical margins is crucial for improving outcomes, underscoring the need for early diagnosis and referral to specialized centers with expertise in complex cardiac tumor resections [36, 37].

Chemotherapy was associated with lower mortality during the early follow-up period, supporting prior studies demonstrating that systemic therapy may improve early disease control and extend survival as part of a multimodality treatment approach [16, 38]. However, this early association diminished over time, likely reflecting the development of tumor resistance, cumulative treatment toxicity, and selection bias favoring healthier patients with greater treatment tolerance [16]. Similar temporal patterns have been reported in prior studies, where the initial efficacy of systemic therapy was followed by relapse or progression due to limited long-term disease control in cardiac sarcomas [37, 39, 40].

Radiotherapy, in contrast, was not significantly associated with primary cardiac malignancy-specific mortality but was linked to modestly lower mortality from other causes, which may reflect supportive-care benefits, improved local symptom control, or advances in modern delivery techniques, such as conformal and intensity-modulated radiotherapy [9, 41]. Although radiotherapy is often used to manage local disease and palliate tumor-related symptoms, durable remission is uncommon, likely due to anatomical constraints, proximity to critical cardiac structures, and limitations in delivering curative doses without cardiotoxicity [42, 43]. These findings are consistent with prior studies indicating that while radiotherapy may improve short-term local control and quality of life, its contribution to long-term survival remains limited in primary cardiac sarcomas [27, 34, 38, 40].

The combination of surgery and chemotherapy was associated with higher overall survival from both rare malignancy and other-cause mortality. Relative to surgery alone, all multimodal combinations were significantly associated with lower mortality from other-cause but showed no significant difference in primary cardiac malignancy-specific mortality. Overall, multimodal strategies may enhance overall survival even when their direct impact on cancer-specific outcomes is limited. The combination therapy is likely to enhance both immediate and sustained survival by targeting residual microscopic disease and reducing recurrence risk [44]. Moreover, potential benefits of radiotherapy are related to improved supportive care or selection of healthier patients for multimodal therapy [34, 40]. Insignificantly association with primary cardiac malignancy-specific mortality may reflect the limited efficacy of radiotherapy in achieving durable tumor control within the cardiac environment due to anatomic and dose constraints [38]. In the cohort of older patients with aggressive disease, the association of multimodal therapy with survival appeared attenuated, suggesting limited potential for substantial long-term improvements in overall survival [40].

Age and disease stage were the most important predictors of survival. Older patients were linked to higher mortality, likely due to comorbidity burden, limited physiological reserve, and lower tolerance for multimodal therapy [34]. Advanced-stage disease was associated with lower survival, highlighting the challenge of delayed detection, while angiosarcoma histology corresponded to less favorable survival, consistent with their clinical behavior reported in previous studies [40, 45].

Importantly, these therapeutic associations were less pronounced among patients aged 65 years and older, a population often characterized by reduced physiological reserve, treatment intolerance, and competing-risk health risks [34, 38]. The attenuation of treatment efficacy in older adults underscores the importance of age-tailored therapeutic strategies, including the use of comprehensive geriatric assessment, individualized dose optimization, and multidisciplinary cardio-oncology collaboration to balance efficacy, safety, and quality of life in this vulnerable population [38, 46]. The RF analysis revealed age, angiosarcoma histology, and cancer stage as the strongest predictors of competing mortality, followed by surgery and chemotherapy, aligning closely with conventional multivariable regression findings [31]. This internal concordance enhances model credibility and underscores the biological plausibility of results [47]. The ML regression tree revealed notable treatment-effect heterogeneity, indicating that younger patients with regional or distant disease were most likely to benefit from surgical therapy, which is aligned with previous oncologic research on sarcoma [32, 48].

The ML survival model showed acceptable discriminative ability and calibration, with a lower mean absolute prediction error, indicating stable performance and internal validity. These findings align with prior work suggesting that ML-based survival methods can accommodate complex, non-linear interactions and high-dimensional data more effectively than traditional regression models [31, 47, 49]. Clinically, these models can support precision oncology by generating individualized survival estimates and guiding shared decision-making for rare malignancies.

This study has several clinical implications. First, early recognition and referral to specialized centers remain crucial, as timely surgical resection was associated with higher survival [34, 35]. Second, chemotherapy was linked to lower early mortality though its long-term associations were less pronounced. Third, ML models may provide complementary information for prognostic assessment, risk stratification, and treatment planning in rare cancer settings [19, 50]. Combining ML with causal inference approaches could help disentangle treatment effects from confounding and better estimate individualized therapeutic benefit. Future studies should focus on external validity and the assessment of model generalizability across multi-institutional or international datasets.

Several limitations warrant acknowledgment. Due to the observational nature of the data, causal associations cannot be inferred. The retrospective design also introduces potential for residual confounding and selection bias. Observed survival differences may be partially influenced by immortal time bias, particularly for chemotherapy and radiotherapy, as patients must survive long enough after diagnosis to receive these treatments. Important clinical variables, including performance status, margin status, and detailed chemotherapy regimens, were unavailable, and comorbidity information was either not recorded or not included in this study. Additionally, sample size constraints limited precision in subgroup analyses, particularly for multimodal treatment combinations and older age groups. Treatment-era effects (early vs late SEER years) were not formally evaluated due to the rarity of primary cardiac malignancies and limited subgroup sizes. Therefore, evolving treatment practices over time, including surgical techniques, chemotherapy regimens, radiotherapy technology, and diagnostic imaging, may have influenced the observed associations. Because of limited treatment detail in SEER, treatments were used in the models as binary indicators of receipt versus non-receipt. This approach does not account for heterogeneity in timing, intensity, or surgical margin status and may lead to residual confounding, misclassification, and attenuation of treatment-related associations. The association of treatment modalities with other-cause mortality may reflect selection bias rather than treatment efficacy. The ML may be subject to overfitting due to the limited sample size and the number of candidate predictors, and that the reported performance metrics should therefore be interpreted cautiously. Moreover, any generalization of these predictive findings requires external validation in independent datasets. Nevertheless, this analysis provides a comprehensive evaluation of treatment outcomes and predictive modeling for one of the rarest and most lethal malignancies.

## Conclusion

Surgical resection, particularly when combined with chemotherapy, was associated with higher survival among patients with primary cardiac malignancies, whereas radiotherapy showed limited association with survival and was primarily related to other cancer-related outcomes. Younger patients with regional or distant disease showed stronger associations between surgical or multimodal approaches and survival, while these associations were attenuated among older adults. These findings underscore the importance of individualized, age-adapted treatment strategies to optimize outcomes in this rare and aggressive cancer.

## Data Availability

The data can be found on the SEER website (https://seer.cancer.gov/). The meta-datasets used and/or analyzed during the current study are available from the corresponding author upon reasonable request.
